# Quantitative Benefit–Risk Assessment: State of the Practice Within Industry

**DOI:** 10.1007/s43441-020-00230-3

**Published:** 2020-10-27

**Authors:** Meredith Y. Smith, Janine van Til, Rachael L. DiSantostefano, A. Brett Hauber, Kevin Marsh

**Affiliations:** 1 Alexion Pharmaceuticals, Inc., 121 Seaport Boulevard, Boston, MA 02210 USA; 2 Department of Regulatory and Quality Sciences, School of Pharmacy, University of Southern California, Los Angeles, CA 90089 USA; 3 University of Twente, Enschede, Netherlands; 4 Janssen R&D, LLC., Titusville, NJ USA; 5 RTI-Health Solutions, Research Triangle Park, NC USA; 6 CHOICE Institute, University of Washington School of Pharmacy, Seattle, WA USA; 7 Evidera, London, UK

**Keywords:** Medicinal products, Medical devices, Structured benefit–risk assessment, Benefit–risk framework, Multi-criteria decision analysis, Discrete choice experiment

## Abstract

**Background:**

Benefit–risk assessments for medicinal products and devices have advanced significantly over the past decade. The purpose of this study was to characterize the extent to which the life sciences industry is utilizing quantitative benefit–risk assessment (qBRA) methods.

**Methods:**

Semi-structured interviews were conducted with a sample of industry professionals working in drug and/or medical device benefit–risk assessments (*n* = 20). Questions focused on the use, timing, and impact of qBRA; implementation challenges; and future plans. Interviews were recorded, transcribed, and coded for thematic analysis.

**Results:**

While most surveyed companies had applied qBRA, application was limited to a small number of assets—primarily to support internal decision-making and regulatory submissions. Positive impacts associated with use included improved team decision-making and communication. Multi-criteria decision analysis and discrete choice experiment were the most frequently utilized qBRA methods. A key challenge of qBRA use was the lack of clarity regarding its value proposition. Championing by senior company leadership and receptivity of regulators to such analyses were cited as important catalysts for successful adoption of qBRA. Investment in qBRA methods, via capability building and pilot studies, was also under way in some instances.

**Conclusion:**

qBRA application within this sample of life sciences companies was widespread, but concentrated in a small fraction of assets. Its use was primarily for internal decision-making or regulatory submissions. While some companies had plans to build further capacity in this area, others were waiting for further regulatory guidance before doing so.

**Electronic supplementary material:**

The online version of this article (10.1007/s43441-020-00230-3) contains supplementary material, which is available to authorized users.

## Introduction

Over the past two decades, both the science and practice of benefit–risk assessment have been subjected to heightened scrutiny from regulators, health technology assessment (HTA) agencies, and manufacturing authorization holders (“sponsors”) of medicinal products and devices. Commensurate with this, a range of initiatives has been launched, including projects aiming to identify applicable benefit–risk assessment frameworks and methods [1–5], catalog relevant graphics and visualization tools for use in benefit–risk decision-making [1, 6, 7], generate evidence-based recommendations for the conduct of benefit–risk evaluation [8, 9], and incorporate patients’ perspectives into such assessments [10, 11].

In parallel with this work, several regulatory authorities have sought to apply specific frameworks and tools—first in the context of pilot studies [5, 8, 10] and then as an integral part of their review process for new drug and medical device marketing authorization applications [12–15]. The term “structured framework” refers to a systematic and standardized format for presenting the relevant benefit–risk data and the supporting narrative. Examples include the Benefit–Risk Assessment Team (BRAT) framework [2] and the Food and Drug Administration’s (FDA’s) Benefit–Risk Assessment Grid [13]. More recently, regulators have sought to incorporate the patient’s perspective into benefit–risk assessment, as reflected in the release of new guidance and accompanying templates [16–20]. Collectively, these efforts have enhanced the scientific rigor, transparency, and consistency with which regulatory agencies conduct benefit–risk assessments [21–25].

Quantitative benefit–risk assessment (qBRA) is one approach to incorporating patients’ (or other stakeholders’) perspectives into benefit–risk assessment. qBRA refers to the combining of data on product performance and stakeholder preferences to inform the assessment of benefit–risk balance. qBRA requires that quantitative weights for—or tradeoffs between—benefits and risks be *explicitly* defined using either stated preference methods [such as discrete choice experiment (DCE), best–worst scaling (BWS), or thresholding] or methods associated with multi-criteria decision analysis (MCDA) (e.g., swing weighting) [10, 26]. qBRA is contrasted with qualitative benefit–risk assessment, in which benefits and risks are measured quantitatively, but the judgment regarding the overall benefit–risk balance is qualitative in nature (i.e., does not involve combining benefit and risk attributes quantitatively). qBRA is also distinct from semi-quantitative benefit–risk assessment. The latter involves aggregating benefits and risks using *implicitly* determined weights, typically those derived from assumptions made by the research team using the ratio of number needed to treat (NNT) to number needed to harm (NNH), or application of net clinical benefit, for example.

qBRA can be utilized across the medicines development lifecycle from discovery through the post-approval period to inform industry, health authority, and reimbursement decisions [27, 28]. For example, in the medicines development phase, understanding what benefit–risk tradeoffs are important to patients and clinicians can be used to inform the prioritization of medicines in the pipeline on the basis of their expected benefit–risk profiles. The application of qBRA is not necessary for every decision across the lifecycle, but can be useful when the benefit–risk decision is more complex [29]. In straightforward scenarios in which the health care intervention has a high benefit and a low risk (or a low benefit and a low risk), a qualitative assessment or semi-quantitative presentation of the clinical data may suffice to articulate the B-R profile. However, if the medicinal product has both high benefit and a high risk, qBRA is useful for determining if patients consider the high benefits to be worth the risk. Similarly, if the medicinal product has a low benefit and a high risk, qBRA could be applied to determine whether at least some patients would be willing to trade the high risk for a modest benefit such as in the context of a condition with high unmet need.

Regulatory interest in qBRA has heightened markedly over the past decade. The European Medicines Agency’s (EMA’s) Benefit–Risk Methodology Project was established in 2009 to review and assess methods for weighing and combining the benefits and risks of treatment [8]. It concluded that one method in particular, MCDA, could support decision-makers in instances where the benefit–risk balance of treatments was marginal. In recent years, the FDA has offered increasing encouragement to sponsors regarding the application of qBRA by publishing examples of patient preference data supporting approval decisions [16], issuing guidance on how to collect patient preference data [4], and defining when patient preference data could support benefit–risk assessment [15]. Most recently, the EMA’s strategic reflection on “Regulatory Science to 2025” included an objective to expand the role of patient preferences in benefit–risk assessments [30].

The extent to which qBRA methods have been adopted within the biopharmaceutical and medical device industry, however, is less well understood. Indeed, despite expanding literature on benefit–risk assessment [2, 3, 21, 22, 31, 32], most of the published studies in this area have focused on novel, “one-off” applications of benefit–risk assessment frameworks and/or qBRA methods [31]. Notable exceptions to this include two case studies that describe how benefit–risk assessment frameworks and toolkits have been integrated into the drug development process within two different biopharmaceutical companies [21, 22, 24, 33].

These two case studies give rise to such questions as: how are other companies across the industry conducting benefit–risk assessments in practice? To what extent are they doing so consistently and comprehensively across their portfolio and the drug life cycle? What frameworks and quantitative methods are they using to do so? What challenges have they faced in seeking to apply them, and how have they surmounted them? Is qBRA useful to inform decisions at different points in the product lifecycle, and if so, when?

The purpose of this study was to address these questions. Specifically, it sought to characterize whether and to what extent the industry is using frameworks to guide benefit–risk assessments, and to describe the types of quantitative methods and tools they are deploying in benefit–risk analyses. Answers to these questions can help identify both emerging best practices in this field, as well as factors that are impeding wider adoption of such approaches. We hypothesized that both large companies and those who had adopted a structured benefit–risk assessment framework to support decision-making would be more likely to have incorporated quantitative methods within that framework in one or more instances.

## Materials and Methods

Study design, data collection, and data analysis were conducted by a research team consisting of subject matter experts and experienced practitioners in the application of pharmaceutical product benefit–risk assessment methods and qualitative data analysis (RD, BH, KM, MYS, JVT). The study received Institutional Review Board (IRB) approval from the University of Twente’s Faculty of Behavioural, Management and Social Sciences.

### Sample

The study sample was generated based on the research team’s collective knowledge of known experts in the benefit–risk field, and through referrals from professional colleagues within the pharmaceutical and drug devices industry. The initial target list of interviewees was subsequently augmented via a snowballing process during data collection.

### Data Collection

Data were collected predominantly via telephone interview, with the exception of one interview that was completed in person. All interviews were recorded, and were conducted between 01 June and 06 December 2019. Each of the four interviewers (RD, BH, KM, MYS) conducted five interviews on average. The conduct of the interviewers is described in accordance with COREQ guidelines in Supplemental Appendix 2.

### Survey Design

Data were collected using a semi-structured survey consisting of 24 questions. The introductory letter sent to eligible participants included a definition of qBRA. Briefly, qBRA approaches combine clinical data on product performance and stakeholder preferences or relative importance weights on the clinical data to inform the assessment of benefit–risk balance (see Supplemental Appendix 1). All survey items were open-ended, although two items regarding company characteristics included a list of possible response options. Topics addressed included: characteristics of the respondent’s company, the respondent’s role within the company, whether structured benefit–risk assessment methods were embedded within internal processes, whether qBRA methods were used, what the decision-making roles and processes within the company regarding qBRA were, the extent of internal support for qBRA, challenges and lessons learned in applying qBRA, how and to what extent the patient voice was incorporated in qBRA, and what the company’s short- and long-term plans were for incorporating qBRA.

The survey instrument was piloted in two eligible participants, and underwent one round of revisions.

### Data Analysis

Both the study design and analysis were guided by the COREQ guideline for qualitative research (see Supplemental Appendix 2). The qualitative study data underwent thematic analysis consisting of multiple steps. First, interview recordings were transcribed using AmberScript (www.amberscript.com, Amsterdam, The Netherlands), a software program that automatically transcribes audio recordings into text using speech recognition. Each interviewer then reviewed their transcriptions for accuracy and manually corrected any identified errors. Next, the corrected transcripts were coded using ATLAS.ti 8.0, a qualitative coding software. A deductive coding process was applied to identify themes corresponding to the interview topics. Subthemes were coded inductively. After the first five interviews, the preliminary coding framework was reviewed, and some groupings were adjusted to accommodate new themes that had emerged. Once all the interviews were coded, results were reviewed to adjust higher order categories that addressed the use of qBRA, impact, implementation, challenges, and future plans. Challenges with respect to the use of qBRA were divided into four subcategories: value proposition, procedural, methodological, and practical issues. The latter three categories were adapted from prior work on challenges in using preferences in HTA [34].

Consistent with recommended practice for qualitative data, we did not present quantitative summaries (such as counts and proportions) when summarizing the data whenever it was unclear if a question or theme was addressed in each interview. Instead, we provided relative frequencies and used descriptive attributions (e.g., “a few,” “some,” and “most”) to facilitate interpretation of relative patterns (e.g., comparing outcomes with other outcomes, as opposed to within responses) and trends [35, 36]. We defined the term “some” to refer to less than half of the responses, and the term “most” to more than half.

## Results

The characteristics of study participants are reported in Table 1. Representatives from 27 companies were contacted, 20 of whom participated. Twelve respondents were senior-level employees working in the areas of patient safety, epidemiology, or benefit–risk assessment. Companies represented in the sample were predominantly large in size (*n* = 17), and either produced pharmaceutical products exclusively (*n* = 9) or both pharmaceutical and medical devices (*n* = 8).

**Table 1:** Characteristics of Participating Companies and Respondents (*n* = 20).

Respondent ID	Type of Products Company Produces	Size of Company	Does Company Use Structured Benefit–Risk Assessment?	Does Company have a Standard Operating Procedure for Structured Benefit–Risk Assessment?	Has Company used qBRA to Support Product Development?^a^
01	Biotechnology	Medium	Yes	Yes	No
02	Medical devices	Large	Yes	No	Yes^a^
03	Pharmaceuticals	Large	Yes	No	Yes
04	Pharmaceuticals	Large	Yes	Yes	Yes
05	Pharmaceuticals	Large	Yes	Yes	Yes^a^
06	Pharmaceuticals	Large	Yes	Yes	Yes
07	Pharmaceuticals	Large	Yes	Yes	Yes
08	Medical devices	Medium	Yes	Yes	Yes
09	Pharmaceuticals	Large	Yes	Yes	Yes
10	Biotechnology	Large	Yes	Yes	Yes
11	Pharmaceuticals	Large	Yes	Yes	No
12	Pharmaceuticals	Large	Yes	Yes	Yes
13	Pharmaceuticals	Large	Yes	No	Yes
14	Pharmaceuticals	Large	Yes	No	Yes^a^
15	Pharmaceuticals	Large	Yes	Yes	Yes
16	Medical devices	Large	No	No	Yes
17	Pharmaceuticals	Large	Yes	No	Yes
18	Pharmaceuticals	Large	No	No	No
19	Biotechnology	Medium	Yes	Yes	No
20	Pharmaceuticals	Medium	Yes	Yes	No

^a^In some instances, respondents responded to the direct question regarding whether their company had used qBRA by answering “no.” However, later in the survey, they indicated that one or more qBRA methods had been used in some capacity. In those instances, the original response was re-categorized as a “Yes*”

### Use and Impact of Structured Benefit–Risk Assessment Framework

Of the 20 responding companies, 18 (90%) reported that their company had adopted a structured approach to benefit–risk assessment, and 13 (65.5%) had a standard operating procedure (SOP) for conducting structured benefit–risk assessment (Table 1).

#### Impact of Structured Benefit–Risk Assessment

Among the companies that were using a structured approach to benefit–risk assessment, several key impacts were cited. One impact concerned improvements in internal team alignment regarding the product’s benefit–risk profile. This enhanced alignment was credited with facilitating internal decision-making, reducing the number and length of meetings, improving internal communication, and increasing the efficiency with which filing documents were developed. Specifically, greater alignment facilitated decisions regarding whether to proceed with a trial, what dose and/or patient population to target in a trial, and whether to proceed with filing a new drug application. As one interviewee noted, “We’ve had situations in which qualitative benefit–risk assessments have led to late Phase 3 terminations, which are a notoriously difficult situation to be in. But I think it could be argued that this was what needed to happen and was best for patient safety” [ID: 13]. The use of structured benefit–risk assessment was also cited as being instrumental in achieving new drug approval in several instances.

Additionally, the use of a structured benefit–risk assessment framework was viewed as having introduced a more systematic approach to decision-making. As one interviewee noted: “… the few products where we work hand-in-hand with the team to pilot the benefit–risk framework in a qualitative manner really helped the team to understand a systematic approach. I don’t know if I could claim … we were able to influence a regulatory decision … but I can claim that we’ve made [a] minuscule, baby step towards shifting the standard of practice so [that] it's a systematic approach” [ID: 20].

### Use and Impact of qBRA

Most participants reported having used qBRA at some juncture in the product life cycle, in contrast to a few participants who reported that they had not yet applied qBRA at all (Supplemental Table 1). Of those who had applied qBRA, it was only for a small number of assets in most instances (e.g., “I can see maybe two or three” [ID: 3], “10 to 20 percent over the course of my tenure” [ID: 13]).

#### At What Phase of Development is qBRA Used?

Most participants reported using qBRA during Phases 2 and 3 of product development. As one participant explained, “Typically in stage three, because that’s when we understand the benefit–risk problem well enough, and that’s where the funding is large enough to cover preference” [ID: 1]. Some participants reported the post-launch use of qBRA. None reported using it in Phase 1.

#### For What Purpose is qBRA Used?

Most participants cited qBRA as being used to support internal decision-making and marketing authorization approval submissions. Internal use of qBRA included: evaluating target product profiles, understanding how the asset was differentiated from current standard of care, supporting the selection of assets to progress from one phase to another, and testing messaging ahead of regulatory submissions. As one respondent noted, “The purpose of the earliest Phase 2A [aBRA assessment] was to understand … the probability that our early development compound may have an advantage … over the standard of care” [ID: 4].

qBRA was also used to guide benefit–risk planning in preparation for filing for marketing approval. As one respondent observed, “The DCE methodology feeds the content of the value tree, which is part of our benefit–risk planning document. So, if there were certain outcomes that we knew were important to … the stakeholder, then we would ensure that those outcomes were part of the value tree which has been evaluated” [ID: 10].

In the post-marketing phase, qBRA was used to understand product uptake and perform periodic benefit–risk analysis, particularly in light of new safety concerns. As one respondent stated, “In the post-marketing setting, in the Periodic Benefit of Risk Evaluation Report is a whole section on benefit–risk evaluation. We are evolving that section to include more quantitative methods instead of a qualitative statement made by physicians” [ID: 19].

#### qBRA Impact

Reports of qBRA impact were mixed. Some participants reported not yet seeing a qBRA impact, partly because it was difficult to measure impact and they had not yet taken steps to do so. Some reported that conducting qBRA raised awareness of the method or gave the team experience in implementing it. Some participants stated that qBRA had an impact on product development—in particular through supporting product approval—and by improving internal decision-making.

Some participants reported qBRA had an impact on approval decisions. For instance, one respondent mentioned that “[results of a] qBRA had actually overridden the Global Patient Safety’s safety concerns, and were instrumental in the project team moving past those concerns to ultimately seeing that product approved” [ID: 13]. A second respondent noted that “a recent submission had the preference study …discussed in the advisory committee meeting … helping reflect the degree to which patients would be accepting of risks. So, we believe it was definitely helpful there” [ID: 15].

Some participants also reported qBRA having an impact on internal decision-making. First, qBRA provided insight that supports the decision. For instance, one respondent noted that qBRA had been useful for selecting a target product profile: “We have some strategic questions in the disease area for which we … may have an asset. The preference study there was extremely important in framing our thinking of where the focus was for that drug” [ID: 15]. Second, qBRA was credited with having improved the efficiency of the decision-making process. For instance, one respondent noted that “there are a lot of different stakeholders looking at the information and seeing it in a different way. [qBRA] has been really helpful to bring alignment … so, I would say speeding decision-making has been our most easily demonstrable advantage” [ID: 4].

### Implementation of Structured and Quantitative Benefit–Risk Assessment

#### Methods for Benefit–Risk Assessment

In terms of approaches to structured benefit–risk assessment, respondents mentioned using one of the following: all or some aspects of the PrOACT-URL [37], the PhRMA’s Benefit–Risk Assessment Team (BRAT) framework [32], or the FDA’s Benefit–Risk Assessment Grid [38] (Supplemental Table 2). One respondent noted that her company had implemented a combined approach that drew from several main benefit–risk assessment frameworks: “So ours is essentially a hybrid. It draws upon the FDA’s benefit–risk assessment grid and also elements of the PrOACT-URL that the EMA uses, and we have, in addition … a standard grid format which we have as a template. We also have two mandatory visuals that go with it: one is a value tree, and the second one is an effects table” [ID: 02].

Among the range of possible qBRA methods used, respondents mentioned two: MCDA and stochastic multi-criteria acceptability analysis (SMAA). In particular, one respondent noted that “I would say we are expanding SMAA methods. And we more or less use end-user preference information for this” [ID: 16]. Similarly, among the range of preference elicitation methods available, respondents mentioned DCE and swing weighting most frequently. Other less frequently mentioned methods include: analytic hierarchy process (AHP), Best-Worst Scaling (BWS), ranking, health state utilities, and threshold technique.

#### Whose Preferences are Elicited, and How are They Incorporated into the qBRA?

Preferences were most commonly reported as being elicited from physicians. As one respondent noted, “We usually or mostly try to get clinicians and people who practice medicine to join our risk management sessions … so that they can also use their expertise on deciding whether or not a risk seen by us is indeed a risk, and whether or not this outweighs the benefits” [ID: 9]. Preference elicitation from patients was also mentioned. One respondent observed, “We’ve done patient preference studies to assess patients’ benefit and risk tradeoffs, which can then inform the weights that go into the MCDA model” [ID: 13]. The preferences of internal stakeholders, such as product team members, were also used in this regard, as reflected in one respondent’s comment: “It begins with internal stakeholders. And when the internal stakeholders have an opportunity to, to weigh in, so to speak, and visualize their benefit–risk story depending on the situation and the way the conversation emerges, it may become, may, it may be obviously a situation where we need others’ voices to weigh in” [ID: 5].

#### Responsibilities for Implementing Quantitative Benefit–Risk Assessment

Several models emerged regarding how structured benefit–risk assessment approaches, including the use of qBRA methods, were implemented within the company. One model was to have a single, dedicated team designated to drive implementation. As one respondent who had worked in benefit–risk assessment in two different companies noted, “We have a benefit-risk team that was put together … to drive the whole exercise. And it has several stakeholders from everybody, basically around in the company. So there are a lot of other stakeholders involved. But it is led by different people. In one company, it was led more by Clinical and in the other company it was led more by Drug Safety” [ID: 10]. A contrasting approach was to have the benefit–risk assessment activities, including specific methods and tools to use, co-implemented by two or more groups. One respondent noted that implementation was handled by representatives from “a combination of Market Access, and to a certain extent, Epidemiology and Pharmacovigilance” [ID: 6]. A final model mentioned involved a collaboration between a specific internal group in conjunction with another company: “The bulk of the work that goes into these … pilots has been through partner companies that are expert in this area and/or [have been] driven by our Health Economics organization” [ID: 11].

### Challenges and Solutions to the Adoption of qBRA

#### Challenges

In terms of challenges to implementing qBRA methods, responses fell into four categories: value proposition, procedural, methodological, and practical (Table 2). The most frequently reported challenge to using qBRA was an unclear value proposition. This challenge referred to the value of qBRA to inform internal decision-making, as well as the value of qBRA as perceived by health authorities, payers, and other stakeholders to inform their decision-making. One respondent noted that, “The FDA and EMA are not swinging their doors open and saying, ‘Send us your quantitative benefit-risk assessments.’ They’re dabbling in it themselves” [ID: 15].

**Table 2:** Challenges in the Use of Quantitative Benefit–Risk Assessment Methods.

Theme	Individual Code	Illustrative Quote
Value proposition	Uncertain value qBRA outside company	*“The FDA and EMA are not swinging their doors open and saying ‘Send us your quantitative benefit-risk assessments’. They’re dabbling in it themselves.” [ID:15]*
	Uncertain value of qBRA within company	*“There is a feeling that we know all we need to know, and this is just adding some numbers to things that we already know. So, there are some doubters in terms of if the outcomes will really provide any novel insights.” [ID:10]*
	Internal resistance to change	*“..[There is a] tendency to kind of fall back into the same routine of the way we used to do things.”[ID:11]*
Procedural	No internal governance	*“I am going to be really frank, the governance around that decision [to conduct qBRA] is still unclear and that’s part of the challenge… It’s really an evolving area at our company right now.”[ID:1]*
	qBRA is not mandatory	*“I think that’s the bigger problem we are having right now with structured approaches. You know, it is not mandatory … for regulatory filing. So, it’s not being used consistently across industry. It is something… nice to have.” [ID:3]*
	Lack of standard process	*“So, this really involves some collaboration by different departments as each has to contribute something. So just it's not about the methodology, or tools, this is about the whole process. So, I can see there are lots of challenges to fully implement.” [ID:14]*
Methods	No clear definition of qBRA	*“The challenge comes in with the [qBRA] definition. Because when referencing ‘quantitative’ there, it [could be] quantitative around representation of the data themselves, not modulation with patient’s preference… It will depend on what one defines as qualitative and what one defines as quantitative.” [ID:7]*
	Unclear which methods to use	*“I think that we need to come as a community to consensus earlier on what are the prioritized methods, having, you know, twenty three or twenty seven methods out there to choose from is, I think, a little overwhelming.”[ID:2]*
	Acceptability of methods	*“You see that the main challenge is the methodology itself – it’s very easy to manipulate. You can easily calculate math…. That’s the perception.” [ID:9]*
Practical	Funding of BRA studies	*“In order for this stuff [qBRA] to be effective or used more, I should say, we need to figure out how to get the cost down.” [ID:2]*
	Timing of qBRA in relation to drug development	*“The biggest challenge is by the time.… Maybe Phase 2 data will warn you about it, but for the most part, you’re not going to know whether you’re in a place where quantitative benefit-risk is needed until the same time [that] you’re rushing to the submission.” [ID:15]*
	Varied expertise in qBRA	*“As much as we’ve had increased understanding, we still have a lot of people who don’t know what these methods are, don’t know the role, and worry about it.”[ID:15]*

qBRA use was also limited by procedural challenges, such as governance issues—e.g., who owns the decision as to whether to perform it, when to perform it, and what methods or procedures were to be used. Varied methodologic challenges were cited, including the lack of consensus regarding which methods to apply in which circumstances, and distrust regarding the application of weights to benefit–risk data. An important practical challenge cited was that qBRA expertise within a company was quite limited, with only a few experts and a few others who had limited experience using qBRA methods.

### Solutions

During the interviews, there were some proposed solutions or countermeasures mentioned to overcome challenges to adopting qBRA approaches. Several respondents noted that having internal champions—especially senior leaders—was critical to the adoption of qBRA methods within their companies. As one respondent reported, “The single most important factor [to qBRA adoption] is senior leadership and championship of the topic in a concrete and sustainable way over time” [ID: 16]. In this regard, an internal champion could reinforce the value proposition of these approaches as well as support development of a standard operating procedure for qBRA. Offering in-house training, and sharing case study examples of the application of qBRA methods was also cited as a way to accelerate qBRA adoption. Finally, anticipated guidance on qBRA from health authorities and other stakeholders was mentioned as a potential catalyst for qBRA adoption for benefit–risk decision-making purposes. Such guidance could help overcome internal resistance to considering qBRA methods by enhancing understanding regarding the value of qBRA. It could also offer information on qBRA methods and insights regarding regulatory expectations concerning when and how to use it, which could further spur qBRA adoption.

### Future Directions

Plans for the future use of structured benefit–risk assessment and/or qBRA approaches were diverse and wide-ranging. These responses fell into three broad categories: capacity building, integration within internal processes, and application (Supplemental Table 3). In terms of capacity building, the most frequently cited responses were to expand the toolkit and guidance for conducting patient preference studies specifically, to pilot or otherwise seek more experience using qBRA methods, and to enhance the qBRA toolkit more generally. Other respondents mentioned plans to expand the application of qBRA methods from strictly internal use to external use, the intention to build a portfolio of internal cases studies, to instill a qBRA “mindset” within product teams, and to build software to support qBRA analyses.

In regard to integration, respondents cited the intention to implement an SOP for structured benefit–risk assessment, or to expand/modify their existing benefit–risk assessment SOP to accommodate qBRA methods. As one respondent noted, “I envision it [i.e., qBRA] to be something that is integrated into our processes. So, that it is something that’s always at least systematically also considered” [ID: 6]. Others noted the need to embed benefit–risk assessment capabilities within product teams, and to integrate the use of patient preference studies earlier in the product development phase.

In addition, respondents intended to apply qBRA methods to inform portfolio decision-making, use it in earlier phases of development or in the post-marketing period, and incorporate benefit–risk data in shared decision-making. Finally, a subset of respondents stated that there were no current plans to advance the use of qBRA methods within their company—a situation attributed largely to lack of regulatory guidance on this issue. As one respondent noted, “It will stay the same, with the minority of [products affected]. This is not going to change. Not going to change until we know what happens with FDA guidance that’s coming soon. If you give guidance, then I think this will drive a lot of [use of quantitative] benefit-risk assessment” [ID: 9].

## Discussion

To the best of our knowledge, this is the first study to describe the state of the practice of qBRA in companies within the life sciences arena. Notably, of the 20 companies who participated in the study, 17 were large in size, and 18 had adopted a structured approach to benefit–risk assessment. The revised International Council for Harmonisation (ICH)’s M4E guidance (revision 2), which was approved in 2015, may have played a facilitating role in this regard, although no respondent specifically referenced it [4]. The M4E revision set forth a standard format for presenting a product’s benefit–risk assessment, one that incorporated the key elements found in the FDA’s Benefit–Risk Assessment Grid [5]. Thus, companies are required to use this structured format when submitting marketing authorization applications via the electronic Common Technical Document (eCTD).

In support of our initial hypotheses, we found that qBRA was being used, especially within companies who had instituted a structured approach to benefit–risk assessment. It was not utilized within medical device companies in our sample. Notably, however, qBRA methods were being used on only a minority of assets. Although restricted to a small number of products, results showed that qBRA was being used throughout the product development process to support product approval and internal decision-making. Of particular note is the example cited by one respondent in which qBRA data were presented at an FDA Advisory Committee meeting. The product in question was subsequently approved by FDA, and the qBRA analysis was viewed by the respondent as having been a contributory factor in this regard.

The comparatively limited use of qBRA within a company’s portfolio of products, however, is consistent with recommended practice in this area. qBRA methods are most appropriate for complex decision-making, especially when the tradeoffs between benefits and risks are highly preference sensitive [5]. Results, however, suggest that qBRA may be underutilized to some extent, due to a variety of factors—perceived uncertainty regarding the value proposition, lack of consensus regarding which qBRA methods to use in which circumstances and how to implement them internally, and the absence of regulatory guidance in this area. Similarly, respondents being unable to point to an appreciable impact associated with using qBRA methods may reflect the fact that none of the companies surveyed were routinely measuring impact. Nor, to the authors’ knowledge, is there clear consensus regarding how to do so.

Despite the relatively limited usage of qBRA and the host of adoption challenges, there was clear evidence of concerted efforts to build capacity in this area—attesting to growing interest in qBRA and an increasing level of sophistication within companies regarding its application. According to several respondents, past and current participation in public–private partnerships (such as IMI-PROTECT [39] and IMI-PREFER [11]) had influenced these internal qBRA activities—especially decisions regarding the inclusion of patient preferences in benefit–risk decision-making. In particular, as the work of IMI-PREFER reaches completion, and their empirically supported recommendations regarding patient preference use in benefit–risk assessment and HTA approval are released, further uptake of qBRA methods should be expected. Additionally, the forthcoming guidance from the FDA’s Center for Drug Evaluation and Research (CDER) and the anticipated EMA guidance on patient preference use in medicinal product benefit–risk assessment will also provide much-needed clarity regarding regulatory perspectives and expectations regarding the incorporation of qBRA in product approval decision-making [5, 13, 30]. Additionally, as more high-quality case studies illustrating the value of qBRA become available, adoption of qBRA methods may accelerate accordingly.

## Limitations

Several study limitations are worth noting. While we included a definition of “qBRA” in the invitation e-mail sent to potential study participants, and again at the beginning of each interview, some respondents may have conflated “structured benefit–risk assessment” (including qualitative and semi-qualitative approaches) with “qBRA” methods. During interviews, efforts were made to clarify respondents’ answers when we detected the potential for such a conflation, such as when they were describing challenges and opportunities to the adoption of specific benefit–risk methods. Despite this, however, discrepancies in respondents’ frame of reference and interpretation of the term “qBRA” may have persisted, and, as a result, may have introduced some ambiguity in our results. For example, a handful of respondents said they did not apply qBRA when questioned initially, but later in the interview provided examples of how they had used qBRA. To correct for this inconsistency, we recoded responses to the question ‘Has your company used qBRA to support product development?’ in Table 1 to ‘Yes*’ if the respondent said ‘No’, but later provided qBRA examples during the interview.

Second, our study sample was derived using the professional networks of the study co-authors, and included respondents who were predominantly from large pharmaceutical and device companies. Hence, it was biased towards those companies and individuals within them, who have been actively engaged in advancing the science of benefit–risk assessment, whether in the form of participation in professional working groups, presentations at scientific congresses, publication in peer-reviewed journals, or some combination of the above. We did attempt to supplement our initial set of companies through a snowballing technique, but only one of the 20 study participants was identified by such means. As a result, our results may not be generalizable to smaller companies and those without an active presence in benefit–risk professional circles.

Lastly, it was difficult to identify who was the single “right” individual within each company to contact for interviewing purposes. The conduct of benefit–risk assessment has been described as a “team sport”—one that demands cross-functional collaboration. As our study results indicated, the responsibility for (and practice of) benefit–risk assessment was often shared across multiple functions or departments within companies, with no one group designated as the “owner” or leader of the process. Future research in this area might benefit by conducting interviews with at least two or three individuals from each respondent company, selected from different parts of the organization, in order to gain a more comprehensive picture of the practice within a given organization.

## Conclusions

Among the life sciences companies we sampled, there was widespread use of qualitative and semi-quantitative benefit–risk assessment approaches. Similarly, many had applied qBRA as well, but its use was concentrated within a small number of assets. The latter finding is consistent with expert guidance recommending that qBRA be used in the context of complex decision-making situations, or in circumstances involving highly preference-sensitive decisions about benefit–risk tradeoffs. Respondents cited several case studies on qBRA impact. Industry investment in capacity suggests an interest in—and potential increased application of qBRA methods in the near future. The anticipated guidance from the FDA’s CDER on qBRA promises to be an important catalyst for its adoption more widely within industry, and its increased application in complex benefit–risk decisions.

## Electronic supplementary material

Below is the link to the electronic supplementary material.

Electronic supplementary material 1 (DOCX 44 kb)

Electronic supplementary material 2 (DOCX 41 kb)

Electronic supplementary material 3 (DOCX 38 kb)

Electronic supplementary material 4 (DOCX 39 kb)

Electronic supplementary material 5 (DOCX 39 kb)

## References

[1] Amarasena IU, Chatterjee S, Walters JA, Wood-Baker R, Fong KM. Platinum versus non-platinum chemotherapy regimens for small cell lung cancer. Cochrane Database Syst Rev. 2015;(8):CD006849 (Epub 2015/08/04). 10.1002/14651858.CD006849.pub3 PMC7263420 26233609

[2] Levitan BS, Andrews EB, Gilsenan A, Ferguson J, Noel RA, Coplan PM (2011). Application of the BRAT Framework to case studies: observations and insights. Clin Pharmacol Ther.

[3] Noel R, Herman R, Levitan B, Watson DJ, Van Goor K (2012). Application of the Benefit-Risk Action Team (BRAT) Framework in pharmaceutical R&D: results from a pilot program. Drug Inf J.

[4] Revision of M4E guideline on enhancing the format and structure of benefit–risk information in ICH, Efficacy: M4E(R2) 2016 (updated 15 June 2016). https://www.ich.org/fileadmin/Public_Web_Site/ICH_Products/CTD/M4E_R2_Efficacy/M4E_R2__Step_4.pdf.

[5] McAuslane N, Leong J, Liberti L, Walker S (2017). The benefit–risk assessment of medicines: experience of a Consortium of Medium-Sized Regulatory Authorities. Ther Innov Regul Sci.

[6] Mt-Isa S, Hallgreen CE, Wang N, Callreus T, Genov G, Hirsch I (2014). Balancing benefit and risk of medicines: a systematic review and classification of available methodologies. Pharmacoepidemiol Drug Saf.

[7] Mt-Isa S, Ouwens M, Robert V, Gebel M, Schacht A, Hirsch I (2015). Structured benefit–risk assessment: a review of key publications and initiatives on frameworks and methodologies. Pharm Stat.

[8] Hughes D, Waddingham E, Mt-Isa S, Goginsky A, Chan E, Downey GF (2016). Recommendations for benefit–risk assessment methodologies and visual representations. Pharmacoepidemiol Drug Saf.

[9] Bollaerts K, De Smedt T, Donegan K, Titievsky L, Bauchau V (2018). Benefit-risk monitoring of vaccines using an interactive dashboard: a methodological proposal from the ADVANCE Project. Drug Saf.

[10] Narita Y, Taniguchi H, Komori K, Kimura K, Kinoshita T, Komori A (2015). Differences in attitude toward adjuvant chemotherapy between colorectal cancer survivors and the medical staff of Japanese hospitals. Int J Clin Oncol.

[11] de Bekker-Grob EW, Berlin C, Levitan B, Raza K, Christoforidi K, Cleemput I (2017). Giving patients’ preferences a voice in medical treatment life cycle: the PREFER Public-Private Project. Patient Patient Cent Outcomes Res.

[12] FDA. Structured approach to benefit–risk assessment in drug regulatory decision-making. FDA; 2013 (updated February 2013). https://www.fda.gov/downloads/ForIndustry/UserFees/PrescriptionDrugUserFee/UCM329758.pdf.

[13] FDA. Benefit–risk assessment in drug regulatory decision-making. FDA; 2018 (updated 30 March 2018). https://www.fda.gov/downloads/ForIndustry/UserFees/PrescriptionDrugUserFee/UCM602885.pdf.

[14] Guidance document on the content of the <Co-> Rapporteur day <60><80> critical assessment report 2011 (Rev. 10.16). https://www.ema.europa.eu/docs/en_GB/document_library/Regulatory_and_procedural_guideline/2016/05/WC500206989.pdf.

[15] Guidance for Industry and Food and Drug Administration Staff: Factors to Consider When Making Benefit–Risk Determinations in Medical Device Premarket Approval and De Novo Classifications. FDA CDRH, 2016 August 30, 2019. Report No.

[16] Ho MP, Gonzalez JM, Lerner HP, Neuland CY, Whang JM, McMurry-Heath M (2015). Incorporating patient-preference evidence into regulatory decision making. Surg Endosc.

[17] Sanft T, Aktas B, Schroeder B, Bossuyt V, DiGiovanna M, Abu-Khalaf M (2015). Prospective assessment of the decision-making impact of the Breast Cancer Index in recommending extended adjuvant endocrine therapy for patients with early-stage ER-positive breast cancer. Breast Cancer Res Treat.

[18] The patient’s voice in the evaluation of medicines. EMA/607864/2013: European Medicines Agency, 2103 EMA/607864/2013 Contract No. EMA/607864/2013.

[19] Postmus D, Richard S, Bere N, van Valkenhoef G, Galinsky J, Low E (2017). Individual trade-offs between possible benefits and risks of cancer treatments: results from a stated preference study with patients with multiple myeloma. Oncologist.

[20] Guidance for Industry and Food and Drug Administration Staff: Patient Preference Information—Voluntary Submission, Review in Premarket Approval Applications, Humanitarian Device Exemption Applications, and De Novo Requests, and Inclusion in Decision Summaries and Device Labeling FDA CDRH, 2016 August 24, 2016. Report No.

[21] Wang J, Wolka A, Bullok K, Anglin G, Radawski C, Noel R (2016). Implementation of structured benefit–risk assessments in marketing authorization applications: lessons learned. Ther Innov Regul Sci.

[22] Wolka A, Warner M, Bullok K, Wang J, Radawski C, Noel R (2016). Incorporation of a benefit–risk assessment framework into the clinical overview of marketing authorization applications. Ther Innov Regul Sci.

[23] Wolka AM, Fairchild AO, Reed SD, Anglin G, Johnson FR, Siegel M (2018). Effective partnering in conducting benefit–risk patient preference studies: perspectives from a patient advocacy organization, a pharmaceutical company, and academic stated-preference researchers. Ther Innov Regul Sci.

[24] Smith MY, Benattia I, Strauss C, Bloss L, Jiang Q (2017). Structured benefit–risk assessment across the product lifecycle: practical considerations. Ther Innov Regul Sci.

[25] Pignatti F, Ashby D, Brass EP, Eichler HG, Frey P, Hillege HL (2015). Structured frameworks to increase the transparency of the assessment of benefits and risks of medicines: current status and possible future directions. Clin Pharmacol Ther.

[26] Guo JJ, Pandey S, Doyle J, Bian B, Lis Y, Raisch DW (2010). A review of quantitative risk–benefit methodologies for assessing drug safety and efficacy—Report of the ISPOR Risk-Benefit Management Working Group. Value Health J Int Soc Pharmacoecon Outcomes Res.

[27] Marsh K, van Til JA, Molsen-David E, Juhnke C, Hawken N, Oehrlein EM (2020). Health preference research in Europe: a review of its use in marketing authorization, reimbursement, and pricing decisions—Report of the ISPOR Stated Preference Research Special Interest Group. Value Health J Int Soc Pharmacoecon Outcomes Res.

[28] Whichello C, Bywall KS, Mauer J, Stephen W, Cleemput I, Pinto CA (2020). An overview of critical decision-points in the medical product lifecycle: where to include patient preference information in the decision-making process?. Health Policy (Amst Neth).

[29] A framework for incorporating information on patient preferences regarding benefit and risk intro regulatory assessments of new medical technology. Medical Device Innovation Consortium; 2015. https://mdic.org/wp-content/uploads/2015/05/MDIC_PCBR_Framework_Web.pdf.

[30] Kazandjian D, Khozin S, Blumenthal G, Zhang L, Tang S, Libeg M (2016). Benefit–risk summary of nivolumab for patients with metastatic squamous cell lung cancer after platinum-based chemotherapy: a Report From the US Food and Drug Administration. JAMA Oncol.

[31] Pinto CA, Tervonen T, Marsh K, Lambrelli D, Schultze A, Tershakovec A (2019). Personalized benefit–risk assessments combining clinical trial and real-world data provide further insights into which patients may benefit most from therapy: demonstration for a new oral antiplatelet therapy. Pharmacoepidemiol Drug Saf.

[32] Coplan PM, Noel RA, Levitan BS, Ferguson J, Mussen F (2011). Development of a framework for enhancing the transparency, reproducibility and communication of the benefit–risk balance of medicines. Clin Pharmacol Ther.

[33] Warner MR, Wolka AM, Noel RA (2016). Implementing benefit–risk assessment for the periodic benefit–risk evaluation report. Ther Innov Regul Sci.

[34] Huls SPI, Whichello CL, van Exel J, Uyl-de Groot CA, de Bekker-Grob EW (2019). What is next for patient preferences in health technology assessment? A systematic review of the challenges. Value Health J Int Soc Pharmacoecon Outcomes Res.

[35] Maxwell JA (2010). Using numbers in qualitative research. Qual Inq.

[36] Neale J, Miller P, West R (2014). Reporting quantitative information in qualitative research: guidance for authors and reviewers. Addiction (Abingdon Engl).

[37] European Medicines Agency Benefit–Risk Methodology Project Work Package 1 Report, EMA/227124/2011 2011 Contract No. EMA/227124/2011.

[38] PDUFA reauthorization performance goals and procedures fiscal years 2018 through 2022. FDA; 2017 (updated 9/1/2011). https://www.fda.gov/downloads/ForIndustry/UserFees/PrescriptionDrugUserFee/UCM511438.pdf.

[39] Nixon R, Stoeckert I, Hodgson G, Pears J, Tzoulaki I, Montero D. IMI WP5 Report 1:b:iv Benefit–Risk Wave 1 case study report: NATALIZUMAB 2013. https://www.imi-protect.eu/documents/NixonetalBenefitRiskWave1casestudyreportNatalizumabMay2013.pdf.

