# Supplementary material for: Quantitative Benefit–Risk Assessment: State of the Practice Within Industry
Source: Ther Innov Regul Sci. 2020 Oct 27;55(2):415–25. doi: 10.1007/s43441-020-00230-3 (PMC7864811; doi:10.1007/s43441-020-00230-3)
Supplement: Supplementary file 1 — Electronic supplementary material 1 (DOCX 44 kb) [file 43441_2020_230_MOESM1_ESM.docx]

# Supplemental Appendix 1: Interview guide

## Introduction

**Background:** Therapeutic benefit-risk assessment is a dynamic and evolving aspect of regulatory science. While structured frameworks have been adopted by regulators in both the US and the EU to guide the benefit-risk assessment process, numerous other issues remain unaddressed. Such issues include, for example, when and how to incorporate preference data, from patients or others, into the benefit-risk assessment? That is, when and how should a quantitative benefit risk assessment (qBRA) be conducted?

Equally important is the question of how industry is responding to this changing regulatory context. To date, little systematic research has been conducted on this topic. Specifically, to what extent have pharmaceutical companies integrated the use of qBRA frameworks to support internal and external decision-making (e.g., with regulatory authorities, with HTA and reimbursement agencies)? How have they organized internally to support this activity (e.g., governance structure, standard operating procedures, staffing and other forms of resource allocation)? What are their future plans in this regard? What role do such factors as company culture, size (dollar revenue), and portfolio type play in this regard? Given that most BRA’s, including qBRAs, require multi-disciplinary collaboration – including expertise in statistics, pharmacoepidemiology, drug safety, risk communication, qualitative research, survey design, preference elicitation, and decision modelling: what resources and expertise are required to effectively implement qBRA, and how have these resources been coordinated to deliver qBRA? Relatedly, how has qBRA been integrated into the methods toolkit?

Lack of clear answers to these questions means that the role of pharmaceutical professionals and other subject-matter experts in delivering qBRA is currently not well-defined. What roles do pharmacovigilance, risk management, drug utilization research, real-world data, and comparative effectiveness research play in qBRA? How do these roles need to interact with other areas of expertise to deliver a qBRA?

**Objectives:** 1. To describe the current state-of-the-art within the industry (i.e., pharmaceutical, medical device and biotechnology companies) in regard to the use of qBRA methods, including quantitative approaches and incorporation of the patient perspective; and, 2) To recommend organizational and governance structures and implementation strategies within sponsoring organizations to enable robust integration of qBRA into internal and external decision-making.

## Information for Participants

**What is the objective this research?** The purpose of this interview is to is to draw on your experience of implementing benefit risk assessment. This interview will take approximately 45 minutes. It will focus on three main categories: 1) characteristics of your company, and its organizational structure; 2) information about how your company is currently conducting benefit-risk assessments (i.e., “state-of-the-art”); and 3) the use of quantitative benefit-risk benefit methods (both current state and future plans).

We define quantitative benefit risk (qBRA) as combining data on product performance and stakeholder preferences to inform the assessment of benefit-risk balance. It requires the explicit definition of quantitative weights for or trade-offs between benefits and risks. This usually involves the use of stated preference methods (such as discrete choice experiment, best worst scaling, or thresholding) or methods associated with multi-criteria decision analysis (MCDA) (such as swing weighting) to elicit stakeholder preferences. qBRA is contrast with:

1. ‘Qualitative BRA’ in which performance on benefits and risks are measured quantitative, but the judgement about the overall benefit-risk balance does not involve quantitative combination of benefit and risk attributes.
2. ‘Semi-quantitative BRA’ in which benefits and risks are aggregated using weights, but the weights are defined implicitly, often based on assumptions made by the research team. For instance, ratio of number needed to treat to number needed to harm, and some applications of net clinical benefit.

We will use the information from this interview to identify lessons for how qBRA can be implemented to support product development.

**Who is on the research team?** The research team comprises staff from the following organizations: Amgen, Evidera, Janssen, RTI and the University of Twente

**Who is funding the research?** No funding has been received to support this study.

**How many people will participate in this research?** It is expected that 20-30 people will participate in this study.

**How will your confidentiality and privacy be respected?** The interview will be audio recorded to ensure we capture everything you say accurately. The recording will be transcribed by the research teams and no names will appear in the written

transcript. Only the research team will be able to access the recordings and transcripts. All personnel accessing your records are required to respect your confidentiality at all times.

To ensure your privacy, your name and other directly identifying information will not be requested during the interview and will not be attached to the records of the interview. Instead, you will only be identified by a code.

**What happens if you change your mind?** Your participation in this study is voluntary. You may discontinue your involvement at any time. If you should withdraw from the study, data collected prior to your withdrawal will not be processed as part of the study.

## Interview Guide

### For the interviewer

The purpose of the interview will be to understand and learn the lessons from participants’ experience of implementing qBRA to inform product development.

The remainder of this document provides a guide for the interviews. It is to be used as a guide only. The actual areas of conversation are fluid, and the interviewer may adapt the guide to cover the questions in the amount of time allotted for the interview session.

While probes are included to supplement many of the questions, the aim of the interview is to elicit as much information as possible without too many suggestions. Please use probes only when necessary, particularly in the early portions of the interview.

The interview session will be approximately 60 minutes of speaking and writing. Use the accompanying Excel form to record the participants’ answers.

Notes to the interviewer are formatted as follows: ***Notes to interviewer***

### Interview

My name is **[name]** and I work at **[company]**. I am part of a multi-organization collaboration conducting a study into the implementation of qBRA in pharmaceutical and medical device manufacturers.

First, I want to thank you for taking time from your busy schedule to participate in this interview.

Before we begin, I would like to confirm that you have read the ‘information for participants’ and that you agree to the interview being audio-recorded.

Do you have any questions before we begin?

***START recorder***

This is study ‘qBRA – a landscape assessment’, interview with participant identification (ID) **[insert number here]** at **[time]** on **[date].**

□ Do I have your permission to record this interview?

□ I want to confirm that you read the ‘information for participants’.

**Questions (40-50 minutes)**

Thank you. I would like to start by briefly discussing your role, and your experience of qBRA.

| Questions | Response |
| --- | --- |
| ***BACKGROUND*** | |
| 1. What types of products does your company, or the part of your company that you represent, produce? 2. Pharmaceuticals 3. Devices 4. Both 5. Other (please state) |  |
| 1. Compared to other companies who produce [AS STATED IN PREVIOUS QUESTION], would you describe your company as 2. “Big pharma” 3. “Large biotech” 4. “Biotech” 5. Small medical device 6. Large medical device 7. Other (please state) |  |
| 1. What is your responsibility at your company? |  |
| ***USE OF BENEFIT RISK ASSESSMENT*** | |
| 1. Does your company have a structured approach to conducting benefit-risk assessment? |  |
| 1. If yes, is this reflected in a standard operating procedure? Can you describe this procedure (general only). |  |
| 1. Is this influenced by any particular BRA framework or guidance e.g. PROACT-URL, BRAT, IMI PROTECT? |  |
| 1. Has your company used ***qBRA*** to support product development?   At what phase of development has qBRA been used, and what was the objective of the qBRA at these phases?  **Examples:**   - **Phase 2, to inform decisions to progress an asset to phase 3** - **Phase 3, to inform a regulatory submission.** |  |
| 1. What was your involvement in these studies?   **Prompt: If the interviewee was involved in all these studies:**  Have other studies been conducted by your colleagues? If so, at what phase of development, and what was their objective? |  |
| 1. **(For each phase / purpose described in q 7)**   Were stakeholder preferences incorporated in the qBRA? If so, which stakeholders’ preferences were incorporated? How were these preferences elicited? How were they incorporated?  **Examples:**   - **Patients’ preference elicited using a discrete choice experiment** - **Clinical team’s preferences elicited using a swing weighting exercise**   **Prompt: If this varied between studies, why?** |  |
| ***ROLES AND PROCESSES*** | |
| 1. **(For each phase / purpose described in qu 3 and 4)**   Who is responsible for deciding whether a qBRA is undertaken at your company?  **Prompt: Is this left to the product team, or is this decision centralized?** |  |
| 1. **(For each phase / purpose described in qu 3 and 4)**   Who is responsible for implementing the qBRA?  If multiple departments are involved, what are their different roles?  **Prompt: Is there specialist capacity to support qBRA, such as a BRA team?** |  |
| 1. Is there a team responsible for co-ordinating the delivery of qBRA? If so, (a) in which department does the team reside? (b) what type of professional training / background do they have? (epidemiology, risk management, health economics, outcomes research) |  |
| 1. Do internal procedures provide guidance on: 2. When qBRA should be performed? 3. How qBRA should be performed? 4. Internal responsibilities for delivering qbRA?   If so, what guidance is provided? |  |
| ***EXTENT OF qBRA*** | |
| 1. What proportion of your assets is supported by qBRA? |  |
| 1. If you are able to say, what is your annual budget for qBRA? How many qBRA’s is this expected to cover? |  |
| ***LESSONS*** | |
| 1. What impact has qBRA had on the success of your assets? Could you provide an example of this impact? |  |
| 1. Has the way that qBRA is implemented at your company changed in the past 5 years? How has it changed, and why? |  |
| 1. What challenges / lessons for the implementation of qBRA have you learned from the above experience?   **Prompt: Lessons for:**   - **When / how qBRA is performed** - **The teams / skills required to successfully implement qBRA** - **The way these teams/ skills are organized** - **Responsibilities for leading qbRA** |  |
| 1. What opportunities / circumstances / facilitators were necessary for the establishment of qBRA in your company? |  |
| 1. Do you have experience of implementing qBRA at a company other than your current company?   If yes, where? How did the way qBRA was implemented at your previous company(ies) differ from you experience at your current company? What are the pros/cons of the implementation at your current company and your previous company(ies)? |  |
| ***PATIENT VOICE*** | |
| 1. If not already discussed already, how is the patient voice incorporated into qBRA at your company? |  |
| ***PLANS*** | |
| 1. What are your company’s plans for quantitative benefit-risk assessment in the near future (1-2 years)? |  |
| 1. What are your plans within the longer term (4-5 years)? |  |
| ***OTHER*** | |
| 1. Is there anything else you’d like to share with me that might inform good practice in how qBRA can be delivered by sponsors? |  |

**End of interview**

Well, we are getting toward the end of the discussion. Can you think of anything important we have left out?

Thank you **very much** for your contributions to this project. We appreciate your time.

Thank you and have a good evening/rest of day/etc.

***STOP Recording***
