# Supplementary material for: Quantitative Benefit–Risk Assessment: State of the Practice Within Industry
Source: Ther Innov Regul Sci. 2020 Oct 27;55(2):415–25. doi: 10.1007/s43441-020-00230-3 (PMC7864811; doi:10.1007/s43441-020-00230-3)
Supplement: Supplementary file 2 — Electronic supplementary material 2 (DOCX 41 kb) [file 43441_2020_230_MOESM2_ESM.docx]

# Supplemental Appendix 2 – COREQ Guidelines Table

| No. Item | Guide questions/description | Reported on Page No. |
| --- | --- | --- |
| **Domain 1: Research team and reﬂexivity** |  |  |
| *Personal Characteristics* |  |  |
| 1. Interviewer/facilitator | Which author/s conducted the interview or focus group? | RD, BH, KM and MYS |
| 2. Credentials | What were the researcher’s credentials? E.g. PhD, MD | PhD |
| 3. Occupation | What was their occupation at the time of the study? | Professionals conducting research in contract research organizations (BH, KM) or in the pharmaceutical industry (RD, MYS) |
| 4. Gender | Was the researcher male or female? | In total, the research team consisted of 3 females, and 2 males |
| 5. Experience and training | What experience or training did the researcher have? | All of the researchers have received either training in interviewing and/or have had extensive professional experience in conducting interviews including for MYS and JVT, courses on qualitative research. |
| *Relationship with participants* |  |  |
| 6. Relationship established | Was a relationship established prior to study commencement? | Yes, in some of the instances. However, every effort was made to treat all interviewees with the same degree of professionalism (i.e., we sent each the topic guide prior to the interview, we introduced ourselves, kept small talk to a minimum during the interview) so this did not affect the data. |
| 7. Participant knowledge of the interviewer | What did the participants know about the researcher? e.g. personal goals, reasons for doing the research | The participants were given the reasons for doing the research. |
| 8. Interviewer characteristics | What characteristics were reported about the inter viewer/facilitator? e.g. Bias, assumptions, reasons and interests in the research topic | Reasons for research |
| **Domain 2: study design** |  |  |
| *Theoretical framework* |  |  |
| 9. Methodological orientation and Theory | What methodological orientation was stated to underpin the study? e.g. grounded theory, discourse analysis, ethnography, phenomenology, content analysis | Grounded Theory |
| *Participant selection* |  |  |
| 10. Sampling | How were participants selected? e.g. purposive, convenience, consecutive, snowball | Purposively sampled, supplemented by some snow-balling |
| 11. Method of approach | How were participants approached? e.g. face-to-face, telephone, mail, email | Face-to-face, and by email |
| 12. Sample size | How many participants were in the study? | 20 |
| 13. Non-participation | How many people refused to participate or dropped out? Reasons? | 7 out of 27 contacted |
| *Setting* |  |  |
| 14. Setting of data collection | Where was the data collected? e.g. home, clinic, workplace | At the workplace in the majority of instances; several interviews were conducted on site at professional conferences. |
| 15. Presence of non-participants | Was anyone else present besides the participants and researchers? | No |
| 16. Description of sample | What are the important characteristics of the sample? e.g. demographic data, date | Demographic |
| *Data collection* |  |  |
| 17. Interview guide | Were questions, prompts, guides provided by the authors? Was it pilot tested? | Interview guides were sent to interviewees prior to the interview. The interview guide was pilot tested. |
| 18. Repeat interviews | Were repeat inter views carried out? If yes, how many? | No |
| 19. Audio/visual recording | Did the research use audio or visual recording to collect the data? | Audiotaped |
| 20. Field notes | Were ﬁeld notes made during and/or after the interview? | Yes, after some interviews |
| 21. Duration | What was the duration of the inter views or focus group? | 30-60 minutes |
| 22. Data saturation | Was data saturation discussed? | Yes and reached |
| 23. Transcripts returned | Were transcripts returned to participants for comment and/or correction? | If desired; no adjustments were made by interviewees |
| **Domain 3: analysis and ﬁndings** |  |  |
| *Data analysis* |  |  |
| 24. Number of data coders | How many data coders coded the data? | 1 |
| 25. Description of the coding tree | Did authors provide a description of the coding tree? | Yes |
| 26. Derivation of themes | Were themes identiﬁed in advance or derived from the data? | Derived from the data |
| 27. Software | What software, if applicable, was used to manage the data? | Atlas.ti software version 7.0 and Word 2007 |
| 28. Participant checking | Did participants provide feedback on the ﬁndings? | No |
| *Reporting* |  |  |
| 29. Quotations presented | Were participant quotations presented to illustrate the themes/ﬁndings? Was each quotation identiﬁed? e.g. participant number | Yes. Tables 3-5. |
| 30. Data and ﬁndings consistent | Was there consistency between the data presented and the ﬁndings? | Yes |
| 31. Clarity of major themes | Were major themes clearly presented in the ﬁndings? | Yes |
| 32. Clarity of minor themes | Is there a description of diverse cases or discussion of minor themes? | Yes |
