# Supplementary material for: Quantitative Benefit–Risk Assessment: State of the Practice Within Industry
Source: Ther Innov Regul Sci. 2020 Oct 27;55(2):415–25. doi: 10.1007/s43441-020-00230-3 (PMC7864811; doi:10.1007/s43441-020-00230-3)
Supplement: Supplementary file 3 — Electronic supplementary material 3 (DOCX 38 kb) [file 43441_2020_230_MOESM3_ESM.docx]

Supplemental Table 1. Use and Impact of Quantitative Benefit-risk Assessment Methods

| Theme | Individual Code | Illustrative Quote |
| --- | --- | --- |
| qBRA undertaken for a minority of assets | Proportion of assets for which qBRA is used | *“Probably 10 percent for preferences. Bear in mind that a lot of our projects are line extensions and application to what we already understand from the patient perspective. It's primarily new medical entities are radically novel line extensions where preferences might become critically important right.” [ID: 15]* |
|  |  | *“It's hard to say because I don't know what the total number of our assets are yet but I would, … I'd say it's probably less than 10 percent. You we're sort of at the early days of actually getting this implemented.”[ID: 21]* |
| Purpose of qBRA | Supporting development strategy | *“The purpose of the earliest phase 2A was to understand … the probability that our early development compound may have an advantage … over the standard of care” [ID: 4]* |
|  | Supporting evidence generation strategy | *“the DCE methodology feeds the content of the value tree which is part of our benefit-risk planning document. So if there were certain outcomes that we knew were important to … the stakeholder then we would ensure that those outcomes were part of the value tree which has been evaluated”. [ID: 10]* |
|  | Supporting regulatory strategy | *“to support regulatory strategy, which dose, which patient, …..where is our product showing the …. most favorable benefit risk? In which subgroup at which dose?” [ID: 4]* |
|  | Support post-launch | *“we did a multi-criteria decision analysis….for in-house decision making ….post-approval, and it gave some insights as to why one product is better received than others from a benefit-risk perspective”. [ID: 15]* |
|  |  | *“in the post marketing setting in the periodic benefit of risk assessment report is a whole section on benefit risk evaluation. We are evolving that section to include more quantitative methods instead of a qualitative statement made by physicians.” [ID: 19]* |
| Impact of qBRA | Supported approval decision | *“qBRA have actually overridden the Global Patient Safety's safety concerns and were instrumental in the project team moving past those concerns to ultimately seeing that product approved” [ID: 13]* |
|  |  | *“A recent submission had the preference study …discussed in the advisory committee meeting … helping reflect the degree to which patients would be accepting of risks. So, we believe it was definitely helpful there”. [ID: 15]* |
|  | Supporting internal decisions | *“We have some strategic questions in the disease area for which we …. may have an asset. The preference study there was extremely important in framing our thinking of where the focus was for that drug.” [ID: 15]* |
|  |  | *“it did help with the choice of which dose to put forward into the application” [ID: 6]* |
|  |  | *“There are a lot of different stakeholders looking at the information and seeing it in a different way. [qBRA] has been really helpful to bring alignment …….. So, I would say speeding decision making has been our most easily demonstratable advantage”. [ID: 4]* |
|  |  | *“… align teams and speed decision making……it helps people get aligned on a common set of key messages, which helps writers write the submission more efficiently, which helps communication. It reduces the number of meetings required [and] the time in the meetings”. [ID: 4]* |
