# Supplementary material for: Quantitative Benefit–Risk Assessment: State of the Practice Within Industry
Source: Ther Innov Regul Sci. 2020 Oct 27;55(2):415–25. doi: 10.1007/s43441-020-00230-3 (PMC7864811; doi:10.1007/s43441-020-00230-3)
Supplement: Supplementary file 4 — Electronic supplementary material 4 (DOCX 39 kb) [file 43441_2020_230_MOESM4_ESM.docx]

Supplemental Table 2. Implementation of Structured and Quantitative Benefit-Risk Assessment

| Theme | Individual Code | Illustrative Quote |
| --- | --- | --- |
| Structured benefit-risk assessment and qBRA methods | Structured benefit-risk assessment methods used | *“So ours is essentially a hybrid. It draws upon the FDA's benefit risk assessment grid and also elements of the PrOACT-URL that the EMA uses, and we have, in addition… a standard grid format which we have as a template. We also have two mandatory visuals that go with it: one is a value tree, and the second one is an effects table.” [ID:2]* |
|  | qBRA methods used | *“I would say we are expanding SMAA methods. And we more or less use end-user preference information for this.” [ID:16]* |
| Preference elicitation methods | Preference elicitation methods | *“…prior to the regulatory engagement, our approach has been…to use swing weighting with the team to engage management, to engage the line heads as needed and to use that information as kind of a level one assessment and to determine whether something more rigorous needs to be taken care of.” [ID:13]* |
|  |  | *“We used discrete choice experiment. I would say for our patient preference studies have been more of a mix but I would say that 70-80% of those used DCE. “[ID:13]* |
| Stakeholder preferences | Stakeholder: physicians | *“…we usually or mostly try to get clinicians and people who practice medicine to join our risk management sessions...so that they can also use their expertise on deciding whether or not a risk seen by us is indeed a risk and whether or not this outweighs the benefits.” [ID:9]* |
|  | Stakeholder: patients | *“We've done patient preference studies to assess patients benefit and benefit and risk trade off which can then inform the weights that go into the MCDA model.” [ID:13]* |
|  | Stakeholder: internal (product development team)” | *“… it begins with internal stakeholders. And when the internal stakeholders have an opportunity to, to weigh in, so to speak, and visualize their benefit risk story depending on the situation and the way the conversation emerges, it may become, may, it may be obviously a situation where we need others' voices to weigh in …” [ID:5]* |
| Responsibility | Deciding whether to undertake qBRA | *“…as of today I think it would be a combination of market access and to a certain extent, epidemiology and pharmacovigilance.” [ID:3]* |
|  | Implementing qBRA | *“…the bulk of the work that goes into these…pilots has been through partner companies that are expert in this area and/or driven have been by our health economics organization.” [ID:11]* |
|  | Coordinating development of qBRA | *“We have a benefit-risk team that was put together the [cross-functional team] to drive the whole exercise. And it has a several stakeholders from everybody, basically around in the company. So there are a lot of other stakeholders involved. But it is led by different people- in one company, it was led more by Clinical and in the other company it was led more by Drug Safety.” [ID:10]* |
