# Supplementary material for: Quantitative Benefit–Risk Assessment: State of the Practice Within Industry
Source: Ther Innov Regul Sci. 2020 Oct 27;55(2):415–25. doi: 10.1007/s43441-020-00230-3 (PMC7864811; doi:10.1007/s43441-020-00230-3)
Supplement: Supplementary file 5 — Electronic supplementary material 5 (DOCX 39 kb) [file 43441_2020_230_MOESM5_ESM.docx]

Supplemental Table 3. Future Directions in the Use of Quantitative Benefit-risk Methods

| Theme | Individual Code | Illustrative Quote |
| --- | --- | --- |
| Capacity Building | Develop qBRA mindset within company | *“…long term is to have benefit-risk thinking and mindset. And then being really proactive on this. So I think anticipating future concerns and being ready to do a proper assessment.” [ID:16]* |
|  | Consider other methods and preference data (e.g., including expanding the toolkit for preference studies; acquisition of dedicated qBRA software; applying the IMI-PREFER guidance on patient preference study design and conduct) | *“I think what we'd like to do is continue to stay abreast of developments of both the EMA and FDA and enhance the toolkit, …, our internal company tool kit. And that's what we're working on this year. It is to come up with some guidance, internal guidance, about the array of different methodologies and approaches that could also be considered depending on the molecule, the timeline, or whatever. So, so we're, so we're, we continually … view ourselves as a continuously learning organization in that regard.” [ID:2]* |
|  | Get more experience using qBRA (e.g., go from exclusively internal to external use; build portfolio of case studies; pilot use of patient experience data in BRA) | *“We have on the horizon additional discrete choice experiments that are planned. We have an initiative around visualization of benefit-risk where I've got one of my statisticians dedicated to the benefit-risk team to help create new ways of looking at quantitative data or quantitative information or quantitative information for the benefit-risk in the asset development teams. So, I know where we're definitely… increasing our involvement and our investment in these methodologies.”[ID:11]* |
| Integration within Internal Processes | Embed the capability for qBRA within the [product] teams’ (e.g., build within company alignment on the purposes of qBRA; provide internal guidance on use of different preference methods) | *“I envision that [i.e., qBRA] to be something that is integrated into our processes. So, that is something that's always at least systematically always considered.” [ID:7]* |
|  | Implement a BRA SOP or modify an existing one | *“And ... as development [of our BRA SOP] further matures, then, the expectation is that the EMA's framework also will be used. The effects' table.” [ID:5]* |
| Application | Include BRA in submission for market approval | *“One would be to shift the standard of practice such that the use of the qualitative framework is consistent across all product marketed products particularly in our periodic reports and that we see the use of that framework in every submission.” [ID:20]* |
|  | Include patient preferences in the [product] design phase | *“… one plan is to include the patient preferences into the design stage of the program, you know, when they are still in the early stage such as the endpoint and population selection. Of course, in the filing stage, if it's applicable, we want to try it.” [ID:14]* |
|  | Have results of qBRA inform decision-making within the company | *“…. bringing the development managers and the managers from patient access area and commercial together …[to] discuss how we can build the capability really together. And, also clarify what the situations are where we can use preference studies for decision making and development and for decision making with HTA parties and payers.” [ID:2]* |
|  | Use qBRA in earlier phases of development | *“… to experiment with its use and application and … in the near term you know trying to find different stages of product development to conduct the research in and then apply it … the focus right now and next stage is mostly in the clinical trial design.” [ID:3]* |
|  | Get advice from [regulatory] authorities and/or health technology assessment (HTA) bodies | *“And… there's some idea in trying to reach out to both HTA and regulatory authorities early on to discuss with them some of those ideas, but I'm not sure how advanced our regulatory and HTA bodies are in terms of having some typic advice, on some, some early discussions.” [ID:6]* |
|  | Use of BRA data in shared decision-making in post-market period | *“I also envision this kind of data being applied in shared decision making in a … post-market setting. I think in a post-market setting, that's probably where this data, the use of this data is more helpful, is in the design of shared decision making tools that can, that are, that are data base data driven, that can ensure easier and more appropriate decision making conversations or processes for patients as they evaluate alternative treatments.” [ID:3]* |
|  | Perform qBRA in most relevant situations | *“I'm looking forward to seeing more BRATs established for projects in the pipeline at phase two and at phase 3. And seeing them execute and seeing a benefit risk profile come out of that effort.” [ID:1]* |
| No plans | No changes to SOP on BRA expected | *“It will stay the same, with the minority of [products affected]. This is not going to change. Not going to change until we know what happens with FDA guidance that's coming soon. If you give guidance, then I think this will drive a lot of [use of quantitative] benefit-risk assessment.” [ID:10]* |
